# Bleeding is associated with intravenous immunoglobulin and therapeutic plasma exchange use in heparin‐induced thrombocytopenia: A propensity matched analysis

**DOI:** 10.1002/jha2.211

**Published:** 2021-05-07

**Authors:** Alexandre Soares Ferreira Júnior, Stephen H. Boyle, Maragatha Kuchibhatla, Oluwatoyosi A. Onwuemene

**Affiliations:** ^1^ School of Medicine Barretos School of Health Sciences Dr. Paulo Prata Barretos São Paulo Brazil; ^2^ Department of Medicine Faculdade de Medicina de São José do Rio Preto (FAMERP) São José do Rio Preto São Paulo Brazil; ^3^ Duke University School of Medicine Durham North Carolina USA; ^4^ Department of Biostatistics and Bioinformatics Duke University School of Medicine Durham North Carolina USA; ^5^ Division of Hematology Department of Medicine Duke University School of Medicine Durham North Carolina USA

**Keywords:** heparin‐induced thrombocytopenia, intravenous immune globulin, National Inpatient Sample, therapeutic plasma exchange

## Abstract

Intravenous immunoglobulin (IVIG) and therapeutic plasma exchange (TPE) are used in select cases with heparin‐induced thrombocytopenia (HIT). In a cross‐sectional analysis, a propensity matched sample was generated by IVIG or TPE treatment status to assess the primary outcome of mortality. In 500 HIT cases, IVIG or TPE was not associated with increased mortality (OR = 1.46; 95% CI: 0.81–2.63, *p* = 0.2052) but was associated with a higher likelihood of major bleeding (OR = 1.75; 95% CI: 1.03–2.96, *p* = 0.0376). The use of IVIG or TPE in HIT cases with bleeding contraindications to standard therapies should be further investigated.

## 1 INTRODUCTION

In selected patients with the immune‐mediated thrombotic disorder, heparin‐induced thrombocytopenia (HIT), treatment includes the use of intravenous immunoglobulin (IVIG) and/or therapeutic plasma exchange (TPE) [1, 2]. IVIG and TPE have been used in severe HIT syndromes characterized by new or progressive thrombosis, limb ischemia, and clinical deterioration [3], and have been demonstrated to improve platelets counts and decrease platelet activation [4, 5]. Due to limited data, recent HIT guidelines include recommendations for TPE prior to cardiopulmonary bypass surgery but give no recommendations regarding use of IVIG [1]. There is little guidance regarding use of either therapy for severe HIT or in patients with contraindications to nonheparin anticoagulant therapy [1, 2, 6]. In a prior analysis of discharge data from the National Inpatient Sample, Healthcare Cost and Utilization Project, Agency for Healthcare Research and Quality [7], we determined that, after controlling for age and Elixhauser comorbidity scores, both IVIG and TPE treatment in HIT were associated with increased mortality and bleeding complications [8]. One challenge in using observational datasets is treatment selection bias in the study design and hence the nonrandom nature of the treatment assignment. This means that the treatment status may be confounded with a number of baseline variables that could be causally related to the outcome. Propensity score analysis is one method used for accounting for such imbalance by matching discharges receiving treatment to nontreated discharges that have similar characteristics, and hence reduce the effects of confounding [9]. Therefore, in the current study, we sought to further investigate our findings with a propensity‐matched analysis.

## 2 METHODS

The study was reviewed by the Duke University Health System Institutional Review Board and determined to be exempt. The study design is a retrospective cross‐sectional analysis of HIT cases in the National Inpatient Sample database from 2010 to 2014. We analyzed discharge data of hospitalized adult patients (≥18 years old) with a primary or secondary diagnosis of HIT. The International Classification of Diseases, Ninth Revision, Clinical Modification code, 289.84 was used to identify HIT cases and codes 99.71 and 99.14 to identify TPE‐ and IVIG‐treated cases. We generated a propensity score matched sample by IVIG or TPE treatment status based on a variety of patient and hospital characteristics. Propensity scores were generated by regressing treatment status on a variety of baseline variables using logistic regression [9]. The variables in the model predicting the treatment status included age, sex, race, thrombotic complications, elixhauser group score, obesity, renal failure, dialysis, year of discharge, hospital bed size, hospital location/teaching status, and hospital region. HIT discharges receiving IVIG or TPE were matched 1:3 with HIT discharges not receiving those therapies using a caliper restriction of 0.5 on the difference between the estimated logits. Using the propensity score matched sample, tests of associations were examined by chi square for categorical variables and Kruskal–Wallis tests for continuous variable, and multivariable regression models were used to examine the association between IVIG or TPE treatment status and the primary and secondary outcomes. The primary outcome was mortality. Secondary outcomes were major bleeding, hospital length of stay, and charges. Logistic regression models were used for binary outcomes. Negative binomial regression models were used for analysis of length of stay and general linear models for hospital charges. The values for hospital charges were log transformed prior to analysis. For mortality, length of stay, and hospital charges, an additional model was fitted including major bleeding as an adjustment variable.

## 3 RESULTS

The identified number of cases with a primary or secondary diagnosis of HIT was 22,152. Among them, 129 cases received IVIG or TPE. Due to missing data for race and hospital variables, propensity score matching was performed on 20,609 discharges, thereby excluding three that received TPE or IVIG. Of the remaining IVIG or TPE discharges, matches were obtained for 125 of 126 (99.2%). The propensity matched data are displayed in Table 1. Comparing the groups with tests of association yielded nonsignificant differences for all variables, indicating that the matching procedure was successful in creating two similar groups.

**TABLE 1:** Propensity matched data using a one to three comparison

	HIT + IVIG/TPE (N = 125)	HIT alone (N = 375)	Total (N = 500)	*p* value
Age in years at admission				0.5553^1^
N	125	375	500	
Mean (SD)	58.4 (16.9)	57.6 (16.7)	57.8 (16.7)	
Median	60.0	58.0	58.0	
Range	(18.0–90.0)	(18.0–90.0)	(18.0–90.0)	
Sex				0.6787^2^
0: Male	56 (44.8%)	176 (46.9%)	232 (46.4%)	
1: Female	69 (55.2%)	199 (53.1%)	268 (53.6%)	
Race				0.6187^2^
1: White	77 (61.6%)	218 (58.1%)	295 (59.0%)	
2: Black	25 (20.0%)	91 (24.3%)	116 (23.2%)	
3: Other	23 (18.4%)	66 (17.6%)	89 (17.8%)	
Thrombotic complication				0.9162^2^
Absent	75 (60.0%)	223 (59.5%)	298 (59.6%)	
Present	50 (40.0%)	152 (40.5%)	202 (40.4%)	
Elixhauser group				0.8411^2^
0–1	≤10	≤10	≤10	
2–3	30 (24.0%)	86 (22.9%)	116 (23.2%)	
4–5	54 (43.2%)	171 (45.6%)	225 (45.0%)	
>5	38 (30.4%)	113 (30.1%)	151 (30.2%)	
Obesity				0.7736^2^
Absent	107 (85.6%)	317 (84.5%)	424 (84.8%)	
Present	18 (14.4%)	58 (15.5%)	76 (15.2%)	
Renal failure				0.3085^2^
Absent	84 (67.2%)	233 (62.1%)	317 (63.4%)	
Present	41 (32.8%)	142 (37.9%)	183 (36.6%)	
Dialysis				0.5701^2^
Absent	91 (72.8%)	263 (70.1%)	354 (70.8%)	
Present	34 (27.2%)	112 (29.9%)	146 (29.2%)	
Year of discharge				0.9439^2^
2010	13 (10.4%)	48 (12.8%)	61 (12.2%)	
2011	31 (24.8%)	86 (22.9%)	117 (23.4%)	
2012	39 (31.2%)	120 (32.0%)	159 (31.8%)	
2013	21 (16.8%)	57 (15.2%)	78 (15.6%)	
2014	21 (16.8%)	64 (17.1%)	85 (17.0%)	
Hospital bed size				0.9210^2^
Small (1–49)	≤10	23 (6.1%)	*	
Medium (50–99)	30 (24.0%)	84 (22.4%)	114 (22.8%)	
Large (>100)	88 (70.4%)	268 (71.5%)	356 (71.2%)	
Hospital location/teaching status				0.8044^2^
Rural	≤10	≤10	≤10	
Urban/Nonteaching	26 (20.8%)	77 (20.5%)	103 (20.6%)	
Urban/teaching	98 (78.4%)	292 (77.9%)	390 (78.0%)	
Hospital region				0.6557^2^
Northeast	22 (17.6%)	54 (14.4%)	76 (15.2%)	
Midwest	29 (23.2%)	82 (21.9%)	111 (22.2%)	
South	52 (41.6%)	179 (47.7%)	231 (46.2%)	
West	22 (17.6%)	60 (16.0%)	82 (16.4%)	

Report generated on November 30, 2020.

^1^ Kruskal–Wallis test.

^2^ Chi‐square.

* The HCUP data use agreement prohibits the reporting of fewer than 11 observations. Cells with frequencies that low are shown as ≤10. Total frequencies for conditions or procedures with one cell with a frequency count of ≤10 are left blank.

Logistic regression analysis of the primary outcome showed a nonsignificant association between the IVIG or TPE group and in‐hospital mortality (OR = 1.46; 95% CI: 0.81–2.63, *p* = 0.2052) and the association remained nonsignificant after adjustment for major bleeding (OR = 1.37; 95% CI: 0.76‐2.49, *p* = 0.2932; Tables 2A and 2B). Analysis of the secondary outcomes showed that cases that received immunomodulatory therapy had a higher likelihood of major bleeding (OR = 1.75; 95% CI: 1.03–2.96, *p* = 0.0376). The association between IVIG or TPE and GI bleeding was positive but not statistically significant (OR = 1.93; 95% CI:0.96‐3.88, *p* = 0.0647). Compared to discharges that received none, IVIG or TPE was associated with a longer hospital length of stay (25.92 vs. 16.44 days, *p* < 0.0001), and more than twice the difference in total hospital charges ($215,147 vs. $96,640, *p* < 0.0001). These associations remained significant after adjusting for major bleeding.

**TABLE 2A:** Regression models evaluating the association of IVIG/TPE to outcomes

	OR (95% CI), *p*‐value	OR (95% CI), *p*‐value*
Major bleeding	1.75 (1.03–2.96), *p* = 0.0376	
GI bleeding	1.93 (0.96–3.88), *p* = 0.0647	
Mortality	1.46 (0.81–2.63), *p* = 0.2052	1.37 (0.76– 2.49), *p* = 0.2932

* Adjusted for major bleeding.

**TABLE 2B:** LSMEANS (95%CI) for analysis examining the association of IVIG/TPE treatment status to hospital length of stay and total charges*

	IVIG/TPE (No)	IVIG/TPE (Yes)		IVIG/TPE (No)**	IVIG/TPE (Yes)	
	LSMEANS (95%CI)	LSMEANS (95%CI)	*p*‐value	LSMEANS (95%CI)	LSMEANS (95%CI)	*p*‐value
Length of stay	16.44	25.92	<0.0001	16.26	25.49	<0.0001
	(15.00–18.01)	(22.17–30.30)		(14.85–17.79)	(21.85–29.74)	
Total charges	96,640	215,147	<0.0001	97,607	208,750	<0.0001
	(85,698–108,979)	(174,520–265,230)		(86,642–109,959)	(169,537–257,032)	

* LSMEANS (95% CI) are presented as exponentiated values.

** Adjusted for major bleeding complications.

## 4 DISCUSSION

Our propensity‐matched analysis evaluating outcomes of HIT cases demonstrated that IVIG or TPE‐treated cases had increased rates of major bleeding without a strong likelihood for mortality.

Bleeding is a known complication of HIT, occurring in up to 50% of cases [10]. As demonstrated in a prior analysis of the Nationwide Inpatient Sample, bleeding is three times more likely to occur in HIT cases compared to non‐HIT cases (6.2 vs. 1.9%; *p* < 0.0001) and in fatal compared to non‐fatal HIT cases (14.7 vs. 5.2%) [11]. Therefore, our study findings of an increased rate of major bleeding in IVIG or TPE‐treated cases indicates that these therapies are most likely to be used in bleeding patients for whom nonheparin anticoagulant therapy is contraindicated. This finding is corroborated by reports in the literature showing use of these therapies in patients with hemorrhage or severe HIT syndromes [3, 12, 13]. In such patients who cannot receive standard nonheparin anticoagulant therapy, outcomes would be expected to be worse compared to HIT cases that tolerate anticoagulation. Therefore, the absence of a significant mortality difference between the matched groups may suggest that use of IVIG or TPE prevents excess mortality in HIT cases that would otherwise have more severe outcomes.

A propensity matched analysis, as was done in this study, accounts for imbalances by matching discharges receiving the treatment to nontreated discharges that have similar characteristics, increasing the probability that the analytic groups are comparable. Thus, we balanced covariates between the comparison groups, including the presence of thrombosis, to yield a control group of non‐IVIG or TPE‐treated HIT cases. This adjustment may account for our findings of no mortality difference between IVIG‐ or TPE‐treated and nontreated HIT cases, which is different from prior nonmatched studies showing increased mortality in HIT cases treated with IVIG (57% vs. 35%; *p* = 0.002) [14] and IVIG or TPE (OR = 1.64; 95% CI: 1.004–2.67, *p* = 0.0480) [8]. Given these differences, the possible mortality benefit of IVIG and TPE in bleeding HIT patients with contraindications to therapeutic anticoagulation should be evaluated prospectively.

The strengths of our study include the application of a propensity matched analysis and use of validated HIT International Classification of Diseases, Clinical Modification‐9 codes with high sensitivity and specificity (90.9% and 94.4%, respectively) [11]. Study limitations include the use of retrospective observational data in an administrative claims database and the absence of granular details of HIT diagnosis, treatment, and temporal relationships between outcomes. Actual outcomes of IVIG or TPE in HIT are best analyzed using large prospective studies.

## CONFLICT OF INTEREST

The authors declare no conflict of interest.

## AUTHOR CONTRIBUTIONS

Alexandre Soares Ferreira Júnior drafted the paper. Stephen H. Boyle and Maragatha Kuchibhatla performed the statistical analysis. Oluwatoyosi Onwuemene designed the research study. All authors revised the manuscript critically and approved the final submitted version.

## Data Availability

The data that support the findings of this study are openly available from Healthcare Cost and Utilization Project (HCUP), Agency for Healthcare Research and Quality, Rockville, MD at http://www.hcup‐us.ahrq.gov/nisoverview.jsp [7].
